# Structural analysis and insight into effector binding of the niacin-responsive repressor NiaR from *Bacillus halodurans*

**DOI:** 10.1038/s41598-020-78148-x

**Published:** 2020-12-03

**Authors:** Dong Won Lee, Young Woo Park, Myung Yeon Lee, Kang Hwa Jeong, Jae Young Lee

**Affiliations:** 1 Department of Life Science, Dongguk University-Seoul, Ilsandong-gu, Goyang-si, Gyeonggi-do 10326 Republic of Korea; 2 Present Address: Structural Biology Lab, B2SBIO, Yeonsu-gu, Incheon, Republic of Korea

**Keywords:** Biochemistry, Molecular biology, Structural biology

## Abstract

The niacin-responsive repressor, NiaR, is transcriptional repressor of certain nicotinamide adenine dinucleotide (NAD) biosynthetic genes in response to an increase in niacin levels. NAD is a vital molecule involved in various cellular redox reactions as an electron donor or electron acceptor. The NiaR family is conserved broadly in the *Bacillus/Clostridium* group, as well as in the Fusobacteria and Thermotogales lineages. The NiaR structure consists of two domains: an N-terminal DNA-binding domain, and a C-terminal regulation domain containing a metal-binding site. In this paper, we report the crystal structures of apo and niacin-bound forms of NiaR from *Bacillus halodurans* (*Bh*NiaR). The analysis of metal-binding and niacin-binding sites through the apo and niacin-bound structures is described. Each N- and C-terminal domain structure of *Bh*NiaR is almost identical with NiaR from *Thermotoga maritima*, but the overall domain arrangement is quite different. A zinc ion is fully occupied in each subunit with well-conserved residues in the C-terminal domain. Niacin is also located at a hydrophobic pocket near the zinc ion in the C-terminal domain.

## Introduction

Nicotinamide adenine dinucleotide (NAD) is an essential molecule that plays an important role as both electron donor and electron acceptor in cellular metabolism^1–3^. The NAD molecule participates in many biochemical transformations, including several reactive centers. Nearly 88% of enzymes that rely on NAD are involved in catabolic pathways. Approximately half of these enzymes are related to the use of carbohydrates, and the remaining enzymes contributes to the oxidation of other substrates such as carboxylic acid, amino acid and fatty acid^4^. In many bacteria, NAD^+^ is synthesized through two metabolic processes, the de novo and salvage pathways. In the de novo pathway, NAD^+^ is synthesized from l-aspartate or l-tryptophan by the consecutive action of NadBACDE (l-aspartate oxidase, quinolinate synthetase, quinolinate phosphor-rybosyl-transferase, nicotinate mononucleotide adenylyl-transferase, and NAD synthetase)^5^. The salvage pathway is a recycling system producing NAD^+^ from exogenous sources such as niacin (also called nicotinic acid or vitamin B_3_), nicotinamide, nicotinamide riboside or endogenous breakdown products of NAD^+^ by NAD^+^-consuming enzymes^1,6^. Despite some variations in the early steps of the two pathways, the final step of NAD^+^ synthesis from nicotinic acid adenine dinucleotide to NAD^+^ is highly conserved^7^.

Both de novo and salvage pathways for NAD^+^ biosynthesis are regulated by several transcription factors, the NAD^+^-dependent repressor (NadR), the Nudix-related transcriptional regulators (NrtR), and the niacin-responsive repressor (NiaR). NadR in Enterobacteria acts as a repressor of certain NAD^+^ biosynthetic genes responding to NAD^+^, nicotinic acid phosphoribosyltransferase (pncB) in the salvage pathway and nadBA involved in the de novo pathway^4^. In addition, NadR has enzymatic activities as nicotinamide riboside kinase and nicotinamide mononucleotide adenylyltransferase, which function in NAD^+^ synthesis^8–11^. The NrtR acts as a regulator of NAD^+^ biosynthesis genes and salvage genes such as, nadBACDE, nicotinamidase (pncA), pncB, and nicotinamide riboside transporter pnuC^12,13^. NiaR, previously called YrxA, was found mainly in *Bacillus/Clostridium* species, as well as Fusobacteria and Thermotogales^6,14^. NiaR represses transcription of certain NAD^+^ biosynthetic genes in response to elevated niacin levels. NiaR also represses the expression of the transporter for niacin uptake (niaX, niaY, and niaP), pncA and pncB in the salvage pathway, and two operons, cysteine desulfurases (nifS) and nadBCA, sharing a promoter region, in the de novo pathway^15^. The transcriptomic analysis of *Streptococcus pneumoniae* NiaR showed that NiaR regulated gene expressions, including niaX, pnuC, and nadC, in response to niacin^16^. The biochemical and bioinformatic analyses provided the mechanism of NiaR and the metabolism of NAD^+^ synthesis in *Bacillus subtilis*^6,17^. The NiaR regulon constitutes a transcriptional regulation system of NAD^+^ synthesis in several groups of Gram-positive bacteria^6^.

Previous studies suggested that niacin binds to the 3-histidine domain (3H domain) of NiaR and NiaR belongs to a family of de novo NAD^+^ synthesis pathway regulators^14^. The NiaR from *Thermotoga maritima* (*Tm*NiaR) displayed metal binding and dimeric structure. The NiaR protein is composed of two domains. The N-terminal domain is a DNA-binding domain containing a helix-turn-helix motif and the C-terminal domain is a regulatory 3H domain in which three histidines are well conserved. A nickel ion is occupied in the C-terminal domain of *Tm*NiaR and the function of this domain was assumed to involve binding to small molecules^18^.

**Figure 1:**
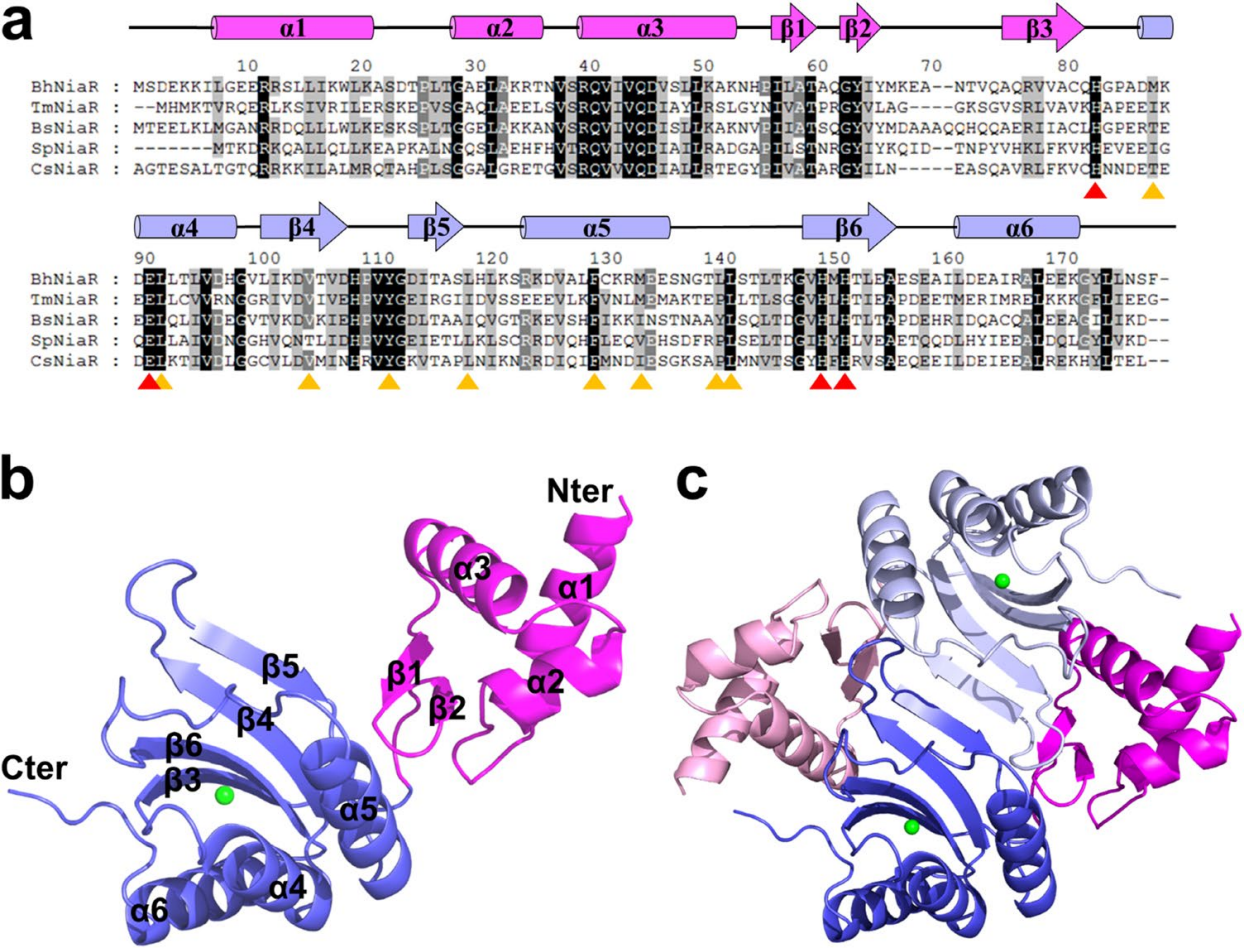
Multiple sequence alignment and overall structure of *Bacillus halodurans* NiaR. (**a**) Multiple sequence alignment of *Bh*NiaR with other NiaR homologues. The secondary structures of *Bh*NiaR are indicated above the sequence. The highly conserved and partially conserved residues are shaded in black and gray boxes, respectively. The residues involved in metal binding are shown as red triangles at the bottom of the sequence. The residues, which form a hydrophobic pocket, are shown as orange triangles at the bottom of the sequence. *Bh*NiaR, *Bacillus halodurans* NiaR; *Tm*NiaR, *Thermotoga maritima* NiaR; *Bs*NiaR, *Bacillus subtilis* NiaR; *Sp*NiaR, *Streptococcus pneumoniae* NiaR; *Cs*NiaR, *Clostridium symbiosum* NiaR. (**b**) The monomeric structure of apo *Bh*NiaR. *Bh*NiaR is composed of an N-terminal DNA-binding domain (magenta) and a C-terminal 3H domain (blue). A zinc ion is shown in green. (**c**) The dimeric structure of apo *Bh*NiaR generated by crystallographic symmetry. The figure was generated using the computer program PyMol (Version 2.3.2, Schrödinger, LLC, https://www.pymol.org).

The NiaR homologue in *Bacillus halodurans* (BH1216, *Bh*NiaR) is composed of 179 amino acids, with 54% sequence identity with NiaR in *B. subtilis* (*Bs*NiaR). Further sequence comparisons of *Bh*NiaR show that it is 37% identical to *Tm*NiaR, 38% identical to *S. pneumonia* NiaR, and 42% identical to *Clostridium symbiosum* NiaR (Fig. 1a).

We determined the crystal structures of *Bh*NiaR with apo and niacin-bound forms. A zinc ion was included in both the apo and niacin-bound models. The presence of the zinc was confirmed by an inductively coupled plasma mass spectrometer (ICP-MS). The structural data of *Bh*NiaR provides details of metal binding and ligand interaction. DNA binding affinity of *Bh*NiaR depending on niacin or metal ions was assessed by electrophoretic mobility shift assay (EMSA).

## Results and discussion

### Model building and quality

The apo crystal structure of *Bh*NiaR was refined to 2.0 Å resolution with crystallographic R_work_ and R_free_ values of 20.18% and 22.78%, respectively. Although the refined model contained a subunit in an asymmetric unit composed of 173 residues with a zinc ion and 129 water molecules, it could be generated to a homo-dimeric structure by a symmetric subunit. The N-terminal domain is composed of residues 7–70 and C-terminal domain is composed of residues 71–179. The N-terminal region (residues 1–6), the linker loop region (residues 68–74) connecting the N- and C-terminal domains, and the internal region (residues 137–147) in the C-terminal domain were poorly ordered due to lack of electron-density maps. The niacin-bound *Bh*NiaR crystals were grown by co-crystallization with 2 mM niacin. The structure of the niacin-bound form was determined at 1.8 Å resolution and refined to crystallographic R_work_ and R_free_ of 19.43% and 24.26%, respectively. The refined model of niacin-bound *Bh*NiaR also contained a subunit with interpretable residues 7–179 in an asymmetric unit. The N-terminus (residues 1–6), the linker loop region (residues 68–74), and the internal region (residues 137–145) in the C-terminal domain were poorly ordered. A niacin molecule was located near a zinc ion and surrounded by a hydrophobic pocket close to the dimeric interface. All refined models for *Bh*NiaR indicated favored or allowed regions of the Ramachandran plot. The detailed refinement statistics are summarized in Table 1.

**Table 1:** Data collection and refinement statistics.

Data set	Apo	Niacin-bound
**Data collection statistics**		
Space group	P4_3_2_1_2	P4_3_2_1_2
Unit-cell parameters		
* a*,* b*,* c* (Å)	42.37, 42.37, 176.2	42.25, 42.25, 176.0
* α*,* β*,* γ* (°)	90.00, 90.00, 90.00	90.00, 90.00, 90.00
Wavelength (Å)	0.97941	0.97941
Resolution (Å)	50.0–2.00 (2.03–2.00)	50.00–1.80 (1.83–1.80)
Number of observations	153,177	111,246
Unique reflections	11,730	15,404
Data completeness (%)	99.9 (100)	97.8 (99.9)
Redundancy	13.1 (13.6)	7.2 (7.9)
Average I/σ(I)	23.21 (9.60)	20.02 (5.98)
R_merge_ (%)^a^	11.1 (40.1)	7.5 (35.2)
**Refinement statistics**		
Resolution (Å)	20.0–2.00	20.0–1.80
R_work_/R_free_ (%)	20.18/22.78	19.43/24.26
No. of non-H atoms	1484	1513
Protein	1354	1354
Ligands	1 (Zn^2+^)	10 (Zn^2+^, Niacin)
Water	129	149
rmsd bonds (Å)	0.003	0.003
rmsd angles (°)	0.513	0.592
Average B-factor	34.68	33.64
Protein	34.41	32.58
Ligands	43.95	33.59
Water	37.47	43.34
Ramachandran plot (%)		
Favored	98.47	98.84
Allowed	1.74	1.16
Outliers	0	0

Values in parentheses refer to the highest resolution shell. ^a^*R*_merge_ = Σ_h_Σ_i_|*I*(*h*)_i_ −  < *I*(*h*) >|/Σ_h_Σ_i_*I*(*h*)_i_, where *I*(*h*) is the intensity of reflection *h*, Σ_h_ is the sum over all reflections, and Σ_i_ is the sum over i measurements of reflection *h.*

### Overall structures of the *B. halodurans NiaR*

Each *Bh*NiaR structure was composed of two domains, an N-terminal DNA-binding domain (residues 1–70) and a C-terminal dimerization/substrate-binding 3H domain (residues 71–179), connected by a flexible loop (residues 66–75) (Fig. 1b). The N-terminal domain contained three α-helices and a pair of anti-parallel β-stands, which form a helix-turn-helix DNA-binding motif. The helix α2 and α3 of the helix-turn-helix motif was generally known as the site of DNA recognition and was well conserved in NiaR homologues. The C-terminal domain contained three α-helices and four anti-parallel β-stands, forming layered α-helices and β-stands of the 3H domain with an order of secondary structural elements in βαββαβα, which is a feature of histidine-containing phosphor-carrier (HPr)-like proteins. Three highly conserved histidines (His83, His150, and His152) and a glutamate (Glu91) form a binding site for the zinc ion and niacin.

A dimeric *Bh*NiaR structure was generated by a crystallographic twofold symmetry (Fig. 1c and Fig. S1). The buried surface area of the dimer is about 1990 Å^2^, approximately 19% of the monomer surface area (PDBePISA protein–protein interaction server: http://www.ebi.ac.uk/msd-srv/prot_int/). The dimeric structure is stabilized by the hydrogen bonds and hydrophobic interactions centered around a faced β5 strand forming an anti-parallel sheet; 22 residues are involved in hydrophobic interactions and 13 residues are involved in hydrogen bonds (PDBsum: http://www.ebi.ac.uk/thornton-srv/databases/pdbsum/Generate.html). The dimer interface is mainly produced by hydrophobic residues, which are highly conserved through NiaR family proteins, such as Val42, Ser48, Leu49, Ala59, Thr60, Ala61, Val78, Val111, Tyr112, Gly113, Ile115, Thr116, Ala117, Ser118, Leu119, Phe130, Met134, Leu141, Val149, Met151, Leu176, and Phe179. Thirteen hydrogen bonds were formed between Glu10 Oε2 and Ser178 Oγ, between Gln41 Nε2 and Thr146 O, between Lys51 Nz and Asp114 Oδ2, between Ala59 N and Asp114 Oδ2, between Ala59 O and Asp114 N, between Tyr64 Oη and Asp108 Oδ2, between Val111 O and Arg133 Nη2, between Asp114 O and Ser118 N, and between Thr116 N and Thr116 O. This finding demonstrated that *Bh*NiaR exists as a functional dimer in solution. A dimer is also consistent with results from size-exclusion chromatography with multi-angle light scattering (SEC-MALS) (Fig. S2). The niacin-bound structure of *Bh*NiaR is almost identical to the apo structure with a root-mean-square deviation (r.m.s.d.) value of 0.2 Å. The most noticeable structural difference was the loop region between the N-terminal and C-terminal domains (Fig. S3).

### Metal-binding site

Previous work suggested Ni^2+^, Cu^2+^, or Zn^2+^ as the most probable metals in the *Tm*NiaR structure based on coordination geometry, anomalous difference Fourier peaks, refinement results, and similar ligating residues in the Metalloprotein Database^14,19^. A zinc ion fully occupied the metal-binding site in the apo and niacin-bound forms of *Bh*NiaR, which was confirmed by ICP-MS and also was verified by a peak on an omit map at the counter levels even at 9σ (Fig. S4). In addition, a nickel ion was partially occupied with *Bh*NiaR (~ 5%). The zinc ion is located between layered α-helices and a β-sheet of the C-terminal domain. The metal-binding site appeared to be fully occupied, with the temperature factors for a zinc ion being 43.95 Å^2^ and 22.81 Å^2^ in the apo and niacin-bound forms, respectively.

**Figure 2:**
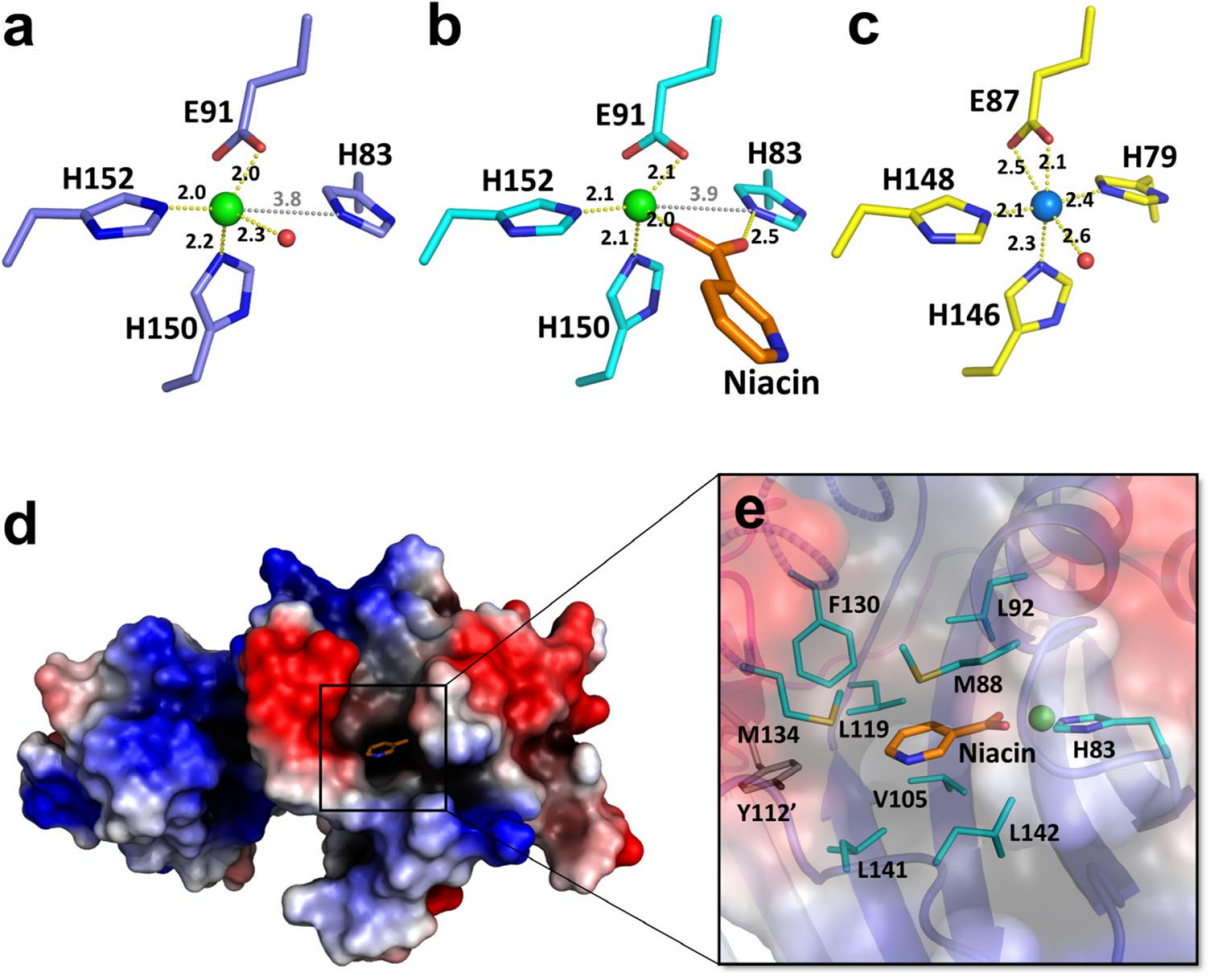
Metal and niacin-binding sites in *Bacillus halodurans* NiaR. (**a**) The metal-binding site of apo *Bh*NiaR. Coordination with the zinc ion (green) and distances between the zinc and the residues (blue) of metal-binding sites are shown in yellow. In addition, a water molecule (red) coordinates with the zinc ion and forms a tetrahedral geometry. In contrast to *Tm*NiaR, the His83 is too distant to coordinate with the zinc ion. The distance between the zinc and His83 is shown in gray. (**b**) The metal-binding site of niacin-bound *Bh*NiaR. The residues are shown in cyan. The niacin (orange) coordinates with the zinc ion instead of the water molecule and interacts with the His83 residue. (**c**) The metal-binding site of apo *Tm*NiaR. Residues of the metal-binding site are shown in yellow. His79 coordinates with the nickel ion (light blue) and forms an octahedral geometry. (**d**) Electrostatic surface diagram of *Bh*NiaR. The blue areas represent positive electrostatic regions and red areas represent negative electrostatic regions. Niacin is bound to the C-terminal domain of *Bh*NiaR. (**e**) Niacin is surrounded by well-conserved hydrophobic residues (cyan) in the C-terminal domain of *Bh*NiaR. The hydrophobic residues make a hydrophobic pocket and van der Waals attractions with niacin (Met88, Leu92, Phe130, Met134, Leu141, Leu142, and Tyr112′). The symmetry-related Tyr112 is colored gray. The figure was generated using the computer program PyMol (Version 2.3.2, Schrödinger, LLC, https://www.pymol.org).

In the apo structure of *Tm*NiaR, it has been reported that four conserved residues (His79, Glu87, His146, and His148) and a water molecule coordinated with a nickel ion in octahedral geometry^14^. However, in the apo structure of *Bh*NiaR, three residues (Glu91, His150, and His152), corresponding to *Tm*NiaR residues (Glu87, His146, and His148), and a water molecule coordinated with a zinc ion in tetrahedral geometry: Glu91 Oε1, His150 Nε1, His152 Nε2, and Wat108 O. The His83 residue was not involved directly in metal coordination, with a distance of 3.8 Å. In addition, in the niacin-bound structure, the coordinated water molecule was replaced by a carboxyl oxygen (O2) of the niacin molecule and the His83 residue formed a hydrogen bond with a second carboxyl oxygen (O1) of the niacin molecule (Fig. 2).

### Niacin-binding site

A niacin molecule is bound to the C-terminal domain of *Bh*NiaR near the metal-binding site. The pyridine moiety of niacin is buried in a hydrophobic pocket surrounded by highly conserved residues (Met88, Leu92, Val105, Leu119, Phe130, Met134, Leu141, Leu142, and Tyr112′) (Fig. 2d,e). The carboxyl group of niacin is located at the position of the water molecule near the zinc ion in apo form. Therefore, the O1 and O2 atoms of the niacin carboxyl group coordinated with His83 Nε2 and the zinc ion, respectively. Coordination of the carboxyl group with the zinc ion and the hydrogen bond with the His83 residue contributed to the stability of niacin binding, as did van der Waals attractions involving nonpolar residues (Met88, Leu92, Phe130, Met134, Leu141, Leu142, and Tyr112′).

### DNA binding affinity

NiaR binds to the promoter region containing the consensus sequence in the presence of niacin^6^. The DNA motif of NiaR proteins is classified into two distinct types. Type I contains the consensus sequence, TGT-N_4_-ACA, characterized in the Fusobacteria lineage and the *Bacillus/Clostridium* groups. Type II contains consensus sequence, ACA-N_5_-TGT, found in the Thermotogales lineage. DNA-binding affinities of type I and II DNA motifs have been investigated by EMSA using NiaR proteins from *B. subtilis* and *T. maritima*^6^.

**Figure 3:**
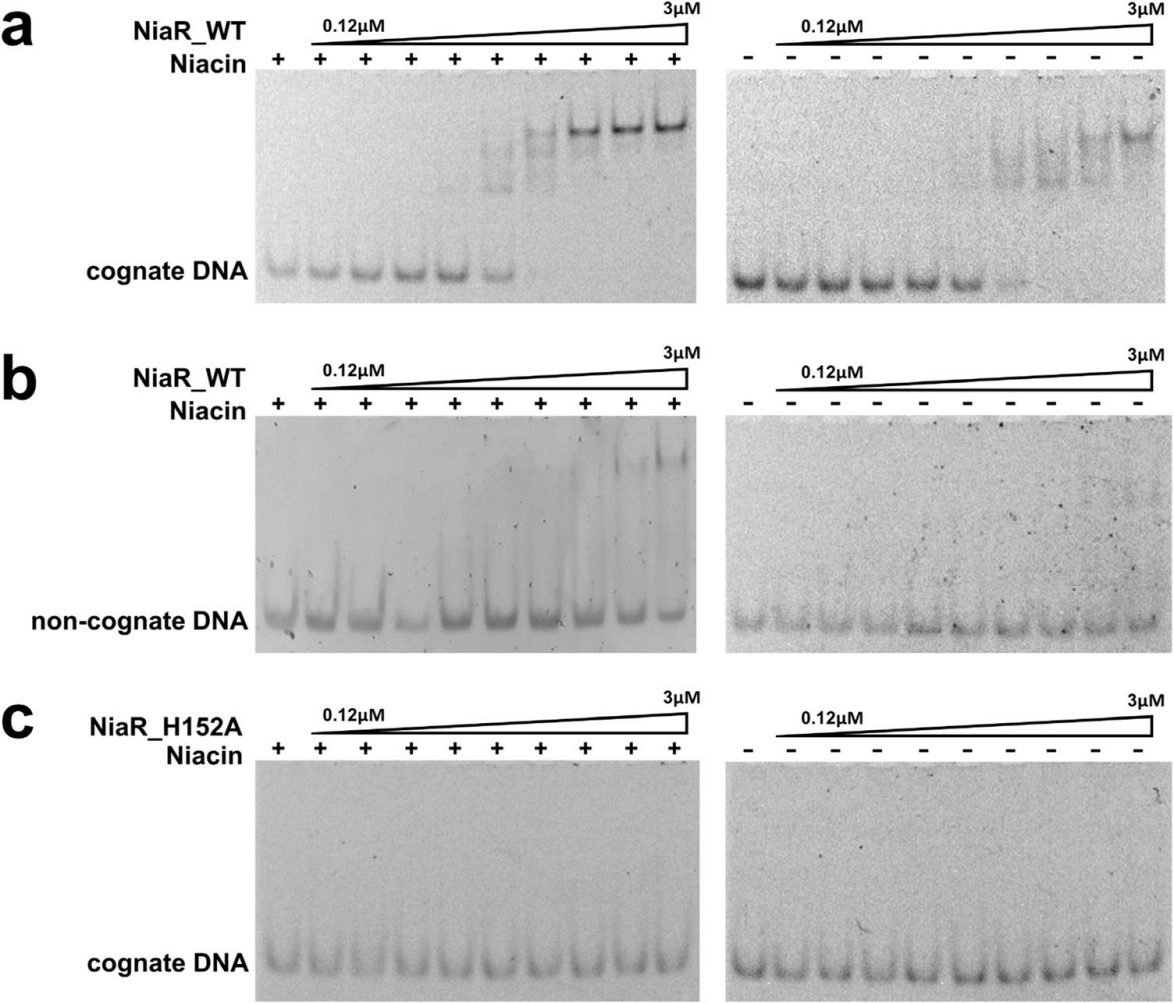
Electrophoretic mobility shift assay (EMSA) of DNA-binding affinity of *Bacillus halodurans* NiaR. The DNA fragments (80 nM) were incubated with *Bh*NiaR (0–3 µM) for 30 min at 277 K with or without niacin (1 mM). The experiments were repeated twice, and representative results are shown. EMSA was performed with the cognate DNA (**a**) and non-cognate DNA (**b**) of *Bh*NiaR. (**c**) The *Bh*NiaR H152A mutant, in which the metal-binding residue is mutated, was incubated with cognate DNA in the presence or absence of niacin. The absence of a zinc ion in *Bh*NiaR caused a significant decrease in the DNA-binding affinity of *Bh*NiaR.

To confirm the DNA-binding affinity of *Bh*NiaR with its predicted cognate DNA segments, we performed EMSA using the type I DNA, 34-bp oligonucleotide (5′–CTCATTTACAT A**TGT**CTTG**ACA**TCTATATTTACA–3′) containing the *nifS-nadB* promoter region. The DNA was mixed with various amount of *Bh*NiaR either in the absence or presence of niacin. *Bh*NiaR bound to its cognate DNA with a dissociation constant (Kd) of ~ 0.5 µM in the presence of niacin, whereas the DNA-binding ability was decreased in the absence of niacin (Fig. 3a). *Bh*NiaR moderately reduced DNA-binding to non-cognate sequences either with or without niacin (Fig. 3b). These results are consistent with homologous NiaR proteins from *B. subtilis* and *T. maritima*^6^.

To further assess whether the metal ion affects the DNA-binding affinity of *Bh*NiaR, a *Bh*NiaR H152A mutant was generated by site-directed mutagenesis resulting in metal-free *Bh*NiaR, which was confirmed by an ICP-MS. The *Bh*NiaR H152A mutant showed a severe defect in DNA-binding affinity regardless of the presence or absence of niacin (Fig. 3c). These results indicate that the metal ion, as well as niacin, plays an important role in DNA binding.

### Structural comparison to other NiaR homologues

The structure of *Bh*NiaR was compared with a NiaR homologue (TM1602) in *T. maritima* (PDB code 1J5Y; r.m.s.d. of 2.2 Å for 108 equivalent Cα positions in residue 63–172 of *Bh*NiaR, a Z-score of 17.7, and a sequence identity of 38%). Although their N- and C-terminal domains were well matched structurally (r.m.s.d. 1.3 Å and 1.2 Å, respectively), the domain arrangements were different. When the C-terminal domains were superimposed, the N-terminal domain of *Bh*NiaR was rotated approximately 170° at the center of the flexible linker region (residues 64–72), compared with that of *Tm*NiaR (Fig. S5). The rotation angle and hinge axes were analyzed using the program Dyndom^20^. The N-terminal domains of *Bh*NiaR were positioned horizontally with their C-terminal domains and the helix-turn-helix motifs were located in the opposite direction. In addition, the protein–protein interaction of the dimer conformation of *Bh*NiaR was different from *Tm*NiaR. *Bh*NiaR formed a dimer between the C-terminal domains as well as between the N-terminal domain of one subunit and the C-terminal domain of the other subunit, whereas *Tm*NiaR formed a dimer through the C-terminal domains alone. The apo and niacin-bound forms of *Bh*NiaR are almost identical. These structural arrangements made it difficult to bind DNA because the N-terminal DNA-binding domains were located at both sides of the C-terminal domains and the helix-turn-helix motifs were not exposed for binding to DNA. The distance between the recognition helices α3 (at residue Arg38) in the *Tm*NiaR dimer was 40 Å, whereas in *Bh*NiaR (at residue Arg40) the distance was 57 Å (Fig. S6). It seems that both the apo and niacin-bound *Bh*NiaR structures display non-DNA bound conformations because of the crystal packing and interactions between the N- and C-terminal domains of each subunit in the dimer. In order to obtain the biologically relevant structure for *Bh*NiaR, the DNA-bound form is required.

## Conclusion

The crystal structures of the niacin-responsive repressor, *Bh*NiaR, which negatively regulates the expression of genes involved in the NAD^+^ synthesis pathway, were determined to be the apo and niacin-bound forms. Our results show the coordination of a zinc ion and the binding of the effector molecule, niacin, in the NiaR structure. In the apo form, the zinc ion is coordinated by three residues (Glu91, His150, and His152) conserved in HPr-like proteins with a water molecule in tetrahedral geometry. Another conserved residue, His83, is not involved in zinc ion coordination but is involved in niacin binding. The niacin bound to the C-terminal domain of *Bh*NiaR is located near a zinc ion, His83, and a well-conserved hydrophobic pocket. Both the niacin and the metal ion cooperatively affect DNA-binding affinity of *Bh*NiaR, and the EMSA results show that the absence of a metal ion causes a significant defect in the DNA-binding affinity of *Bh*NiaR.

## Materials and methods

### Cloning, expression, and purification

The *niaR* genes were amplified by polymerase chain reaction (PCR) using genomic DNA of *B. halodurans* as a template. The amplified *niaR* genes were inserted into the NheI/XhoI-digested expression vector pET-28b(+) (Novagen), resulting in a hexahistidine-containing tag (His-tag) at its N-terminus with a PreScission protease cleavage site. The recombinant *niaR* genes were transformed in *Escherichia coli* BL21 (DE3) star pLysS cells (Invitrogen). The transformed cells were grown at 310 K to an OD_600_ of ~ 0.5 in Luria–Bertani medium and overexpression of *Bh*NiaR protein was induced continuously with 1.0 mM isopropyl *β*-d-1-thiogalactopyranoside (IPTG) for 4 h at 303 K. After the cells were collected by centrifugation at 4200*g* for 10 min at 277 K, the pellets were immediately frozen at 193 K.

The frozen pellets were placed in a lysis solution containing buffer A (20 mM Tris–HCl, pH 8.0, 0.5 M NaCl, 10% (*v/v*) glycerol) and 1 mM phenylmethylsulfonyl fluoride, homogenized by an ultrasonic processor (Sonics Vibra Cell, VCX750) and separated from the insoluble fraction by centrifugation at 31,000*g* for 60 min at 277 K. The recombinant *Bh*NiaR protein in soluble fraction was loaded on a nickel-charged His-trap immobilized metal affinity chromatography (IMAC) column (GE Healthcare, UK) and eluted with buffer B (20 mM Tris–HCl, pH 8.0, 0.5 M NaCl, 10% (*v/v*) glycerol, and 300 mM imidazole). Enzymatic removal of the His-tag from the *Bh*NiaR proteins was achieved by overnight incubation with PreScission protease. The uncleaved His-tagged proteins were removed from the native proteins by applying to a nickel-charged His-trap IMAC. The next purification step was size-exclusion chromatography on a Superdex-75 column (GE healthcare) with elution buffer (20 mM Tris–HCl, pH 8.0, 0.2 M NaCl, 5% (*v/v*) glycerol, 1 mM dithiothreitol and 1 mM MgCl_2_). The purified *Bh*NiaR protein was concentrated to 15 mg ml^−1^ using Centricon YM-10 (Millipore).

### Site-directed mutagenesis

Site-directed mutagenesis of *Bh*NiaR was performed by PCR using a recombinant plasmid containing the wild-type *niaR* gene as a template. Two complementary primers were designed such that the encoded His152 (CAC) was replaced by GCA-encoded alanine (Forward primer: 5′–GTGTTCATATGGCAACGTTAGAAGC–3′, and Reverse primer: 5′–GCTTCTAACGTTGCCATATGAACAC–3′). PCR products were treated with DpnI for digestion of preexisting recombinant plasmids containing the wild-type *niaR* gene. PCR products were introduced into *E. coli* DH5α, and then the mutant plasmids were isolated and purified from the cells. The mutated *niaR* sequence was confirmed by DNA sequencing (Macrogen, Republic of Korea).

### Crystallization and X-ray data collection

Initial crystallization of the *Bh*NiaR protein was performed by the sitting-drop vapor diffusion method using a 96-well crystallization plate (SWISSCI MRC, UK) and commercial screening solution from Hampton Research, Qiagen and Emerald Biosystems at 296 K. Each sitting-drop was prepared by mixing 0.75 μl of the concentrated protein and reservoir solution, respectively. The *Bh*NiaR protein grew as two forms of crystals, apo form and niacin-bound form. Crystals of apo *Bh*NiaR were obtained in 2% (*v/v*) tacsimate, pH 6.0, 0.1 M Bis–Tris, pH 6.5, and 20% (*v/v*) PEG 3350 solution and were grown to 0.05 × 0.1 × 0.05 mm within 2 weeks. Crystals of niacin-bound *Bh*NiaR were obtained by co-crystallization with 2 mM niacin and were grown in 0.1 M Tris–HCl, pH 8.5, and 15% (*v/v*) PEG 6000 solution.

Each crystal was transferred into a cryo-protectant solution containing 20% (*v/v*) glycerol in the reservoir solution and then flash-cooled in liquid nitrogen. X-ray diffraction data of apo and niacin-bound crystals of *Bh*NiaR were collected at 100 K and at the wavelength, 0.97941 Å, using synchrotron radiation of the Pohang Accelerator Laboratory in Korea. Diffraction data were collected with a Pilatus 6 M detector on 11C Micro-MX beamline for the apo form and with an ADSC Q315r CCD detector on 5C-SBII beamline for the niacin-bound form. The crystals were exposed to X-rays for 1.0 s per image, and 270 frames (apo) and 100 frames (niacin-bound) were obtained with each 1.0° oscillation. The data were processed and scaled with *DENZO* and *SCALEPACK* of *HKL2000* software^21,22^.

### Structure determination and refinement

The crystal structures of both forms of *Bh*NiaR were solved by molecular replacement using the program *PHASER MR* from the CCP4 program suite^23^ using the *Tm*NiaR structure (PDB code 1J5Y) as a search model^14^. The structures of apo and niacin-bound forms built by *COOT*^24^ and were refined by *PHENIX*. All refined structures of *Bh*NiaR were evaluated by *MolProbity*^25^ and have been deposited in the Protein Data Bank. The figure was generated using the computer program PyMol (Version 2.3.2, Schrödinger, LLC, https://www.pymol.org)^26^. The refinement statistics of *Bh*NiaR are shown in Table 1.

### Electrophoretic mobility shift assay

To confirm the DNA-binding affinity of *Bh*NiaR, 34-bp double-stranded DNA (Forward : 5′–CTCATT**TACATATGTCTTGACATCTATA**TTTACA–3′ and Reverse : 5′–TGTAAA**TA TAGATGTCAAGACATATGTA**AATGAG–3′) containing own promoter region (*nifS-nadB*) was prepared by Oligo Synthesis (Macrogen, Republic of Korea). The reaction mixture was made by mixing the native *Bh*NiaR (0–3 µM) and the reaction buffer (20 mM Tris, pH 8.0, 100 mM KCl, 1 mM MgCl_2_, 1 mM dithiothreitol, and 5% (*v/v*) glycerol with or without 1 mM niacin) prior to mixing the double-stranded DNA (80 nM). All reaction mixtures were incubated for 30 min at room temperature, after which each mixture was loaded on a pre-chilled 6% native polyacrylamide gel in 0.5 X Tris–Borate, pH 8.3. Electrophoresis was performed at 277 K and the gel was visualized using SYBR Green (ThermoFisher Scientific).

### Size-exclusion chromatography with multi-angle light scattering (SEC-MALS)

SEC-MALS experiments were performed using a fast protein liquid chromatography system (GE Healthcare) connected to a Wyatt DAWN Heleos II instrument and a Wyatt Optilab T-Rex differential refractometer. A Superdex-200 10/300 GL (GE Healthcare) gel filtration column pre-equilibrated with 20 mM Tris–HCl (pH 8.0), 200 mM NaCl, and 1 mM DTT was normalized using ovalbumin (40 kDA) as the protein standard. The *Bh*NiaR protein was injected (10–12 mg ml^−1^, 0.1 ml) at a flow rate of 0.5 ml min−^1^. The data were evaluated using the Zimm model for static light-scattering data fitting and represented using an EASI graph with a UV peak in the *ASTRA V* software (Wyatt).

#### Accession numbers

Coordinate and structure factors have been deposited in the Protein Data Bank (PDB): apo *Bh*NiaR, PDB ID, 7CV0; Niacin-bound *Bh*NiaR, PDB ID, 7CV2.

## Supplementary information

Supplementary Figures.

## Data Availability

All data are fully available without restriction.
