# Supplementary material for: Structural analysis and insight into effector binding of the niacin-responsive repressor NiaR from Bacillus halodurans
Source: Sci Rep. 2020 Dec 3;10:21039. doi: 10.1038/s41598-020-78148-x (PMC7713382; doi:10.1038/s41598-020-78148-x)
Supplement: Supplementary file 1 — Supplementary Figures. [file 41598_2020_78148_MOESM1_ESM.pdf]

Structural analysis and insight into effector binding of the niacin-responsive repressor NiaR from *Bacillus halodurans*

Dong Won Lee[1,3], Young Woo Park[1,2,3], Myung Yeon Lee[1], Kang Hwa Jeong[1], Jae Young Lee[1]*

[1] Department of Life Science, Dongguk University-Seoul, Ilsandong-gu, Goyang-si, Gyeonggi-do, 10326, Republic of Korea

[2] Present address: Structural Biology Lab, B2SBIO, Yeonsugu, Incheon, Republic of Korea

[3] These authors contributed equally.

*Corresponding author

E-mail: jylee001@dongguk.edu

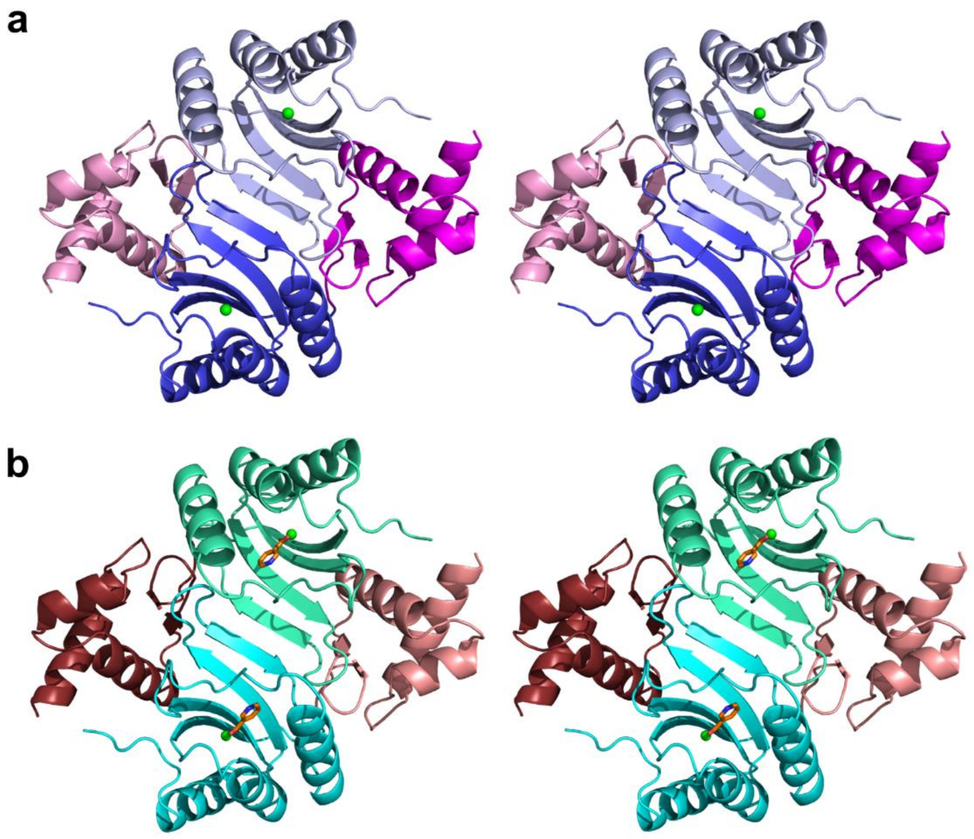

Supplementary Figure 1. Stereo-view of dimeric structure of *Bacillus halodurans* NiaR. (**a**) Dimeric structure of apo *Bh*NiaR. The N-terminal domains are shown in magenta and pink. The C-terminal domains are colored blue and light blue. The zinc ions are indicated in green. (**b**) Dimeric structure of niacin-bound *Bh*NiaR. The N-terminal domains are shown in light brown and brown. The C-terminal domains are colored cyan and green cyan. The niacin is shown in orange.

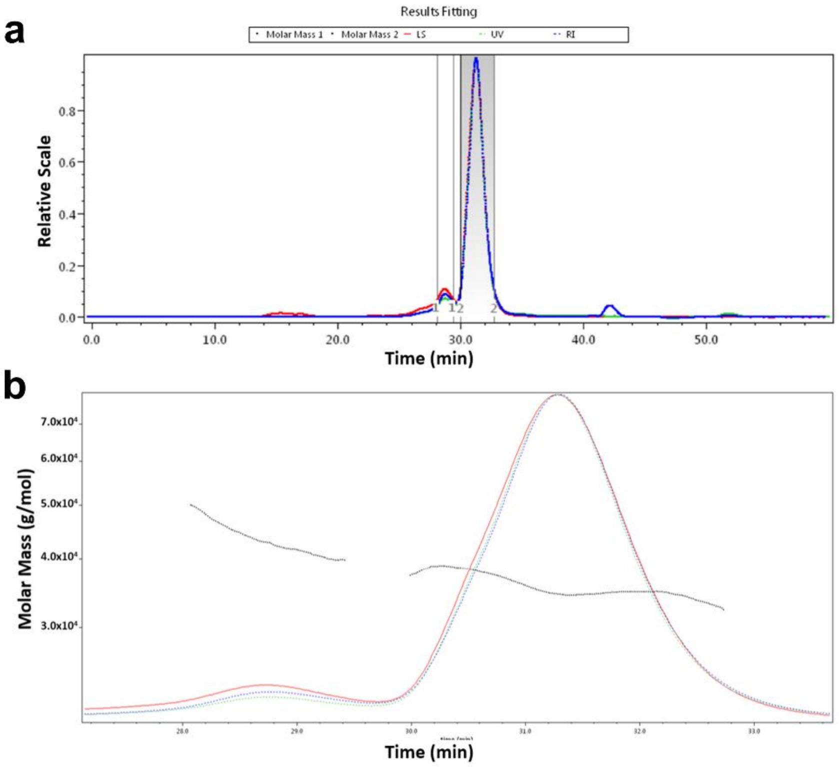

Supplementary Figure 2. Result of size-exclusion chromatography with multi-angle light scattering (SEC-MALS). (**a**) Peak profiles of *Bh*NiaR using SEC-MALS. (**b**) Molar masses of corresponding to the two peaks.

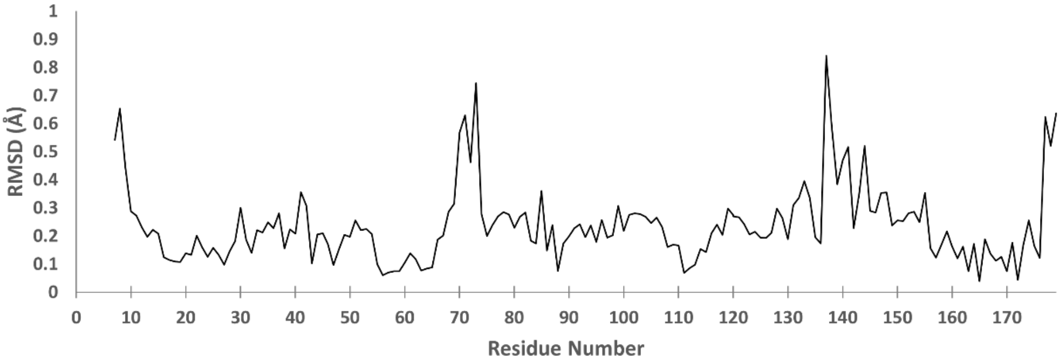

Supplementary Figure 3. R.m.s.d plot between apo and niacin-bound *Bacillus halodurans* NiaR.

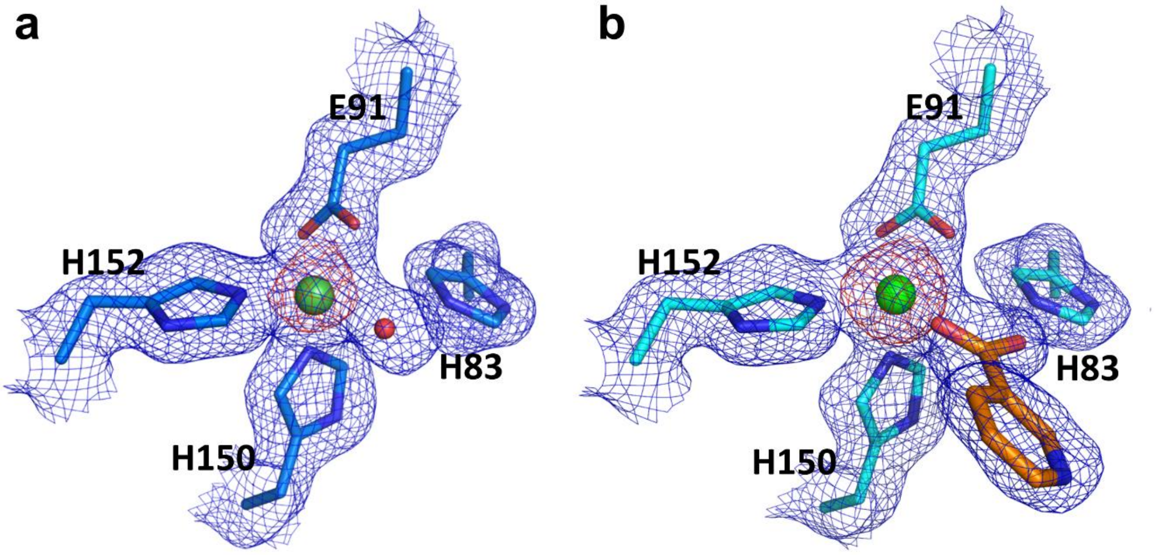

Supplementary Figure 4. Electron density map of metal-binding site in the *Bacillus halodurans* NiaR. (**a**) Electron density map of metal-binding site in apo *Bh*NiaR. A σA-weighted electron density map (2Fo-Fc map) contoured at 1.0σ (blue). An omit map was calculated, contoured at 5σ (red). The zinc ion (green) is depicted with surrounding residues (blue). (**b**) Electron density map of metal-binding site in niacin-bound *Bh*NiaR. The coordinating residues are shown in cyan, and niacin is shown in orange. A σA-weighted electron density map (2Fo-Fc map) contoured at 1.0σ (blue). An omit map was calculated, contoured at 5σ (red).

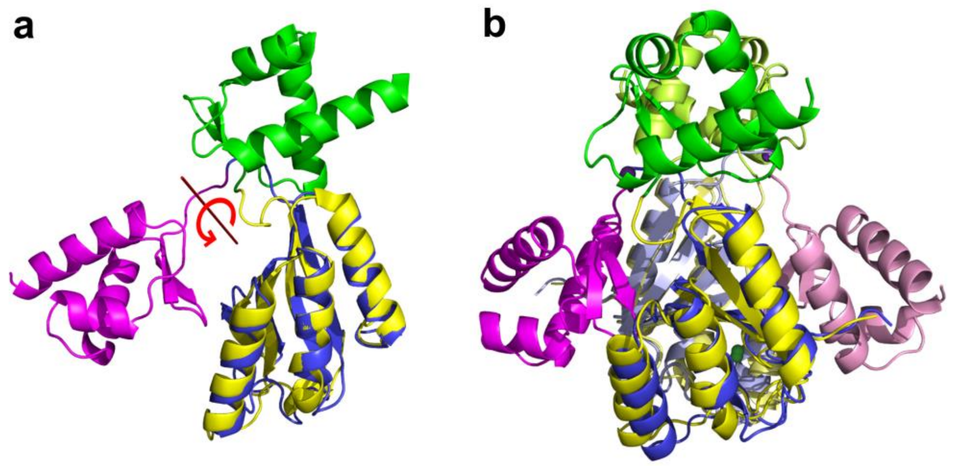

Supplementary Figure 5. Structural comparison between *Bacillus halodurans* NiaR and *Thermotoga maritima* NiaR. (**a**) Superimposition of apo *Bh*NiaR with apo *Tm*NiaR based on C-terminal domain. The N-terminal domain of *Bh*NiaR (magenta) shows approximately 170° rotation compare to *Tm*NiaR (green), against hinge axes colored red. (**b**) Superimposition of dimer *Bh*NiaR with dimer *Tm*NiaR based on C-terminal domain.

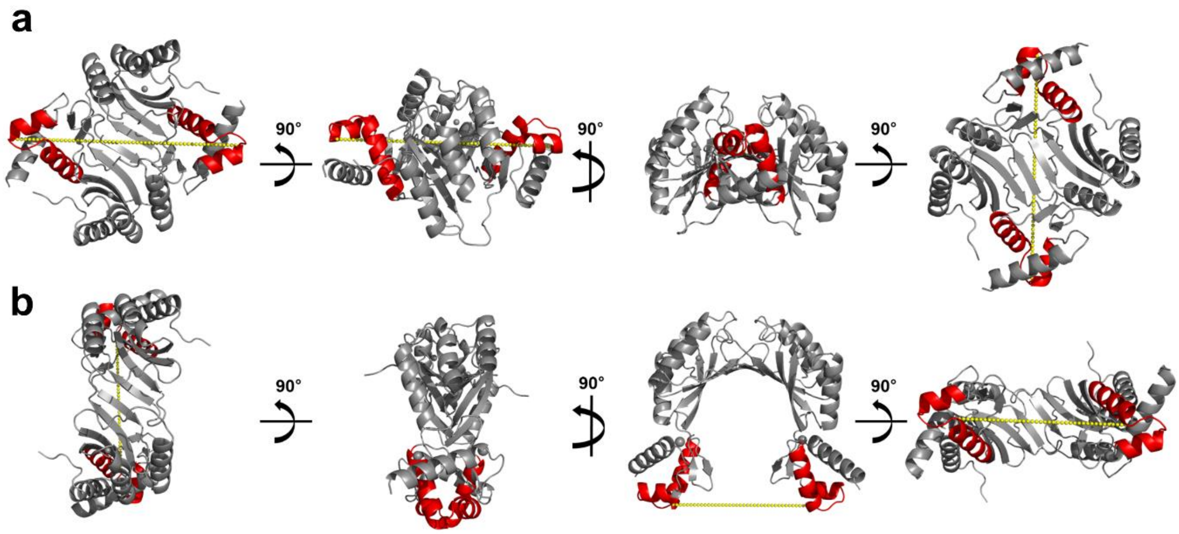

Supplementary Figure 6. The dimeric structures of *Bacillus halodurans* NiaR (a) and *Thermotoga maritima* NiaR (b) with same orientation of the C-terminal domain. The HTH motifs of *Bh*NiaR and *Tm*NiaR are shown in red.

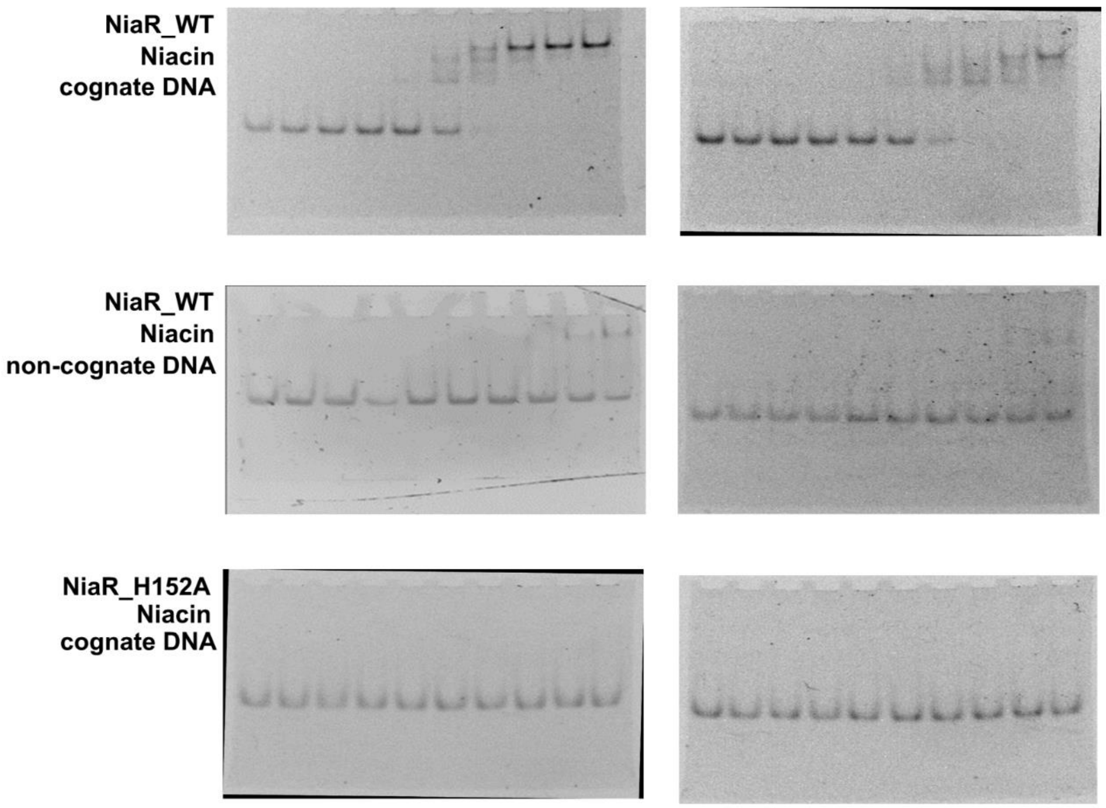

Supplementary Figure 7. Uncropped images of gel shown in Figure 3.
